# Role of Phytoestrogen in Menopausal Women With Depressive Symptoms: A Consecutive Case Series Study

**DOI:** 10.7759/cureus.37222

**Published:** 2023-04-06

**Authors:** Stergios Karalis, Tilemachos Karalis, Foteini Malakoudi, Ioannis Thanasas, Angeliki S Kleisiari, Zacharoula Tzeli, Evie Papavasiliou, Dimitrios T Karalis

**Affiliations:** 1 Department of Internal Medicine, General Hospital of Trikala, Trikala, GRC; 2 1st Department of Obstetrics and Gynecology, Papageorgiou General Hospital of Thessaloniki, Thessaloniki, GRC; 3 2nd Department of Cardiology, Papageorgiou General Hospital of Thessaloniki, Thessaloniki, GRC; 4 Department of Obstetrics and Gynaecology, General Hospital of Trikala, Trikala, GRC; 5 Department of Nutrition and Dietetics, University of Thessaly, Trikala, GRC; 6 Department of Management Science and Technology, University of Western Macedonia, Kozani, GRC; 7 Department of Public Health and Primary Care, University of Cambridge, Cambridge, GBR; 8 Department of Nutrition and Dietetics, University of Thessaly, Volos, GRC

**Keywords:** menopausal symptoms, estrogen replacement therapy, depression prevention, phytoestrogens, menopause

## Abstract

Introduction

During menopause, healthy women experience a diverse aggregate of clinical manifestations and symptoms that relate to hormonal and aging changes. These alterations are strictly associated with psychological disorders, mainly depression. Estrogen treatment may be effective for these mood variations caused by menopause.

Aim

To demonstrate the impact of phytoestrogen treatment in menopausal women with depressive symptoms.

Methods

The study is a consecutive case series study, with a six-month follow-up. It was conducted in a private consultant endocrinologist clinic in Trikala, Greece. A total of 108 eligible participants aged from 45 and above experiencing depressive symptoms were included. The Beck Depression Inventory-II (BDI-II) questionnaire for depressive symptoms was measured at three time points (t=0, t=3 months, t=6 months) and the means were analyzed and compared to each other.

Results

The overall mean BDI-II scores reveal that depressive symptoms constantly and gradually decreased over time, with the numbers of postmenopausal women experiencing minimal or mild depression and moderate depression, as tested at baseline and study completion (before and after phytoestrogen use), being inversely proportional.

Conclusion

Phytoestrogen administration to menopausal women is indicated to reduce depression symptoms. More research in the area is needed to reach definite conclusions.

## Introduction

Menopause, a critical point in female life, defined as the permanent cessation of menstrual cycles induced by the loss of ovarian follicular activity [1], is frequently associated with various concerns and complaints over commonly occurring symptoms, such as mood disturbances, predominantly relating to anxiety and depression [2,3].

There has been increasing evidence suggesting that since the fluctuation or reduction of estrogen levels during menopause may decrease serotonin in the brain and lead to low mood or even depression, estrogens can be effectively used to manage menopause-induced mood disturbances, resulting in hormone replacement therapy (HRT) being largely prescribed to alleviate depressive symptoms in menopausal women [4-6]. The plethora of adverse effects, however, including increased risk of breast cancer, cardiovascular disease, dementia, stroke, and other thromboembolic events, has seriously hampered estrogen use [7,8].

Τo counteract the potential risks reported, the use of phytoestrogens, which are compounds extracted from plants, primarily isoflavones that mimic or modulate endogenous estrogens, has been explored as an alternative approach to managing post-menopausal depression [9]. As a result, herbal medicine has been widely opted for to maintain physical and cognitive health, averting, at the same time, exposure to the risks associated with HRT [10].

The current study seeks to assess and report on the effects that phytoestrogens, in particular isoflavones, may have beneficial use on managing depressive symptoms in healthy postmenopausal women.

## Materials and methods

This is a consecutive case series study conducted to assess the effects of phytoestrogen use on depressive symptoms in postmenopausal women. The study took place at a private consultant endocrinologist clinic in mainland Greece, from March 2020 to November 2021 (National Ethics Committee for Clinical Studies approval 55480/6-09-2006). Postmenopausal women attending the clinic were invited to participate. The Beck Depression Inventory-II (BDI-II) questionnaires, evaluating different psychological symptoms, were assigned to the subjects. Consequent patients of the pre-specified study period were enrolled based on a set of predefined inclusion/exclusion criteria.

Inclusion criteria comprised postmenopausal women, aged 45 or older (the age at which menopause as a natural part of aging usually occurs), having had their last menstruation within a minimum of one and a maximum of three years, and experiencing any vasomotor symptoms, such as hot flashes, sweating, including night sweat, sleep disturbances, anxiety/stress and heart palpitations of any severity and at any frequency. Women were asked to recall and describe their experience of any vasomotor menopausal symptoms over the past four weeks prior to the commencement of the study, drawing from a list of symptoms shown to them, and classify their severity as no/light/moderate/heavy and their frequency as no/once a day/twice a day/three times or more daily, before they were enrolled.

Exclusion criteria involved women using any type of HRT, women with a history of a gynecological condition or past surgery that could provoke an earlier onset of menopause (before 45 years old), and women with a prior diagnosis of mental health or other psychological condition that might have led to receiving psychiatric treatment. Postmenopausal women with thyroid disorders and oncological diseases were also excluded.

**Figure 1:**
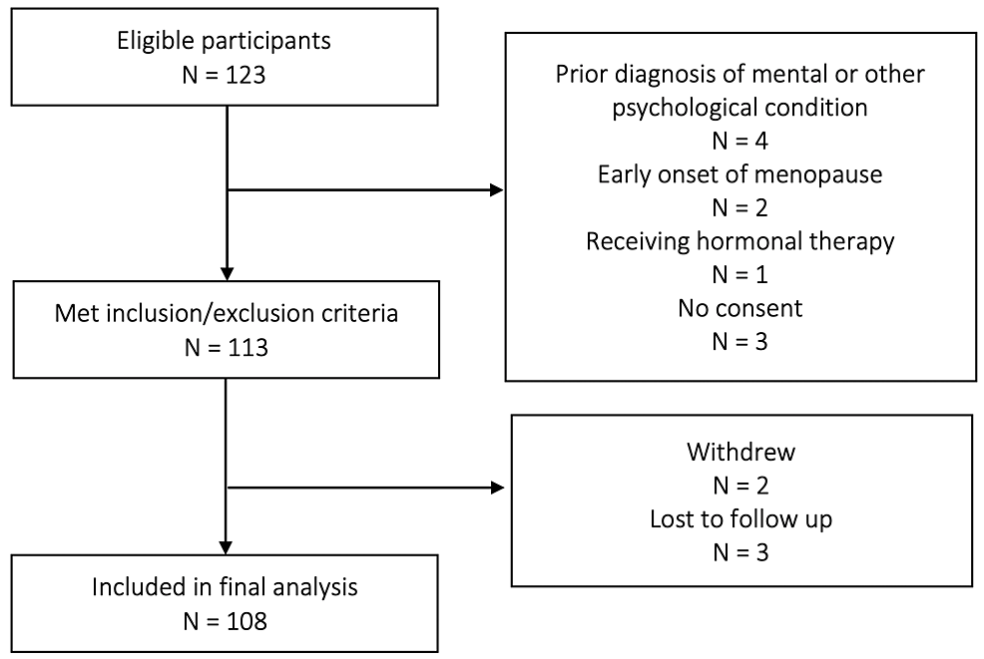
Participant selection flow chart

In total, 123 postmenopausal women were invited to participate and 113 of them met the inclusion and exclusion criteria. In the end, 108 participants were included in the final analysis, after two women withdrew and three were lost to follow-up. The flow diagram for participant selection is shown in Figure 1.

The participants, upon enrolling in the study, completed a written form declaring their age (in years), their gender ("male", "female", "other/please describe", "prefer not to say"), their marital status ("married", "not married"), their smoking status ("never smoked", "current or ex-smoker"), and their alcohol intake ("none", "low", "moderate", "high"). Afterward, body mass index (BMI) was calculated by measuring height using a built-in stature meter in an upright position, without shoes, and with an accuracy of 0.5 cm, and weight in light clothing and without shoes on an electronic precision weighing scale. Measurements were rounded to hundredths. To ensure that BMI was to remain stable throughout the study, customized Mediterranean diet plans were followed to meet the dietary needs of each study participant.

The exposure was deemed to be the administration of phytoestrogen tablets containing 54.4 mg of soy isoflavones. The consistency of every capsule also included calcium (120 mg/cap), vitamin D3 (7.5 μg/cap), melatonin (1 mg/cap), vitamin B6 (1.4 mg/cap), folic acid (200 μg/cap), vitamin B12 (2.5 μg/cap), a-linolenic acid (200-288.9 mg/cap), and magnesium (56.25 mg/cap). They were advised to take it once a day, just before or at bedtime, for a period of six months.

The psychological state of participants at the predetermined time points of the study was quantified using Beck Depression Inventory-II (BDI-II).

Testing started at baseline/prior to the commencement of the study (t = 0) and was repeated in three months (halfway through the study, t = 3) and in six months (upon study completion, t = 6). Consultations for clinical evaluation and assessment were held fortnightly and drop-in review and discussion sessions (with mandatory attendance) occurred the next day of testing. The latter allowed for clarification of any issues or questions the participants may have.

In terms of testing, primary questionnaire-based data were collected via BDI-II, a 21-item self-report instrument that is used to measure/assess major depressive symptoms [11]. Four response options are presented for each item on a scale of 0 to 3. For example, to measure sadness, the response options range from “I do not feel sad” (score of 0) to “I am so sad and unhappy that I cannot stand it” (score of 3). Items were then summed to create a total score, with higher scores indicating higher levels of depression, evaluated as follows: 0-16: minimal or mild depression, 17-30: moderate depression, and 30-63: severe depression.

Simple descriptive statistics were used to analyze the data collected. To measure the change in depressive symptoms ‘before’ and ‘after’ the use of phytoestrogen tablets containing 54.4 mg soy isoflavones once daily and give a sense of scale and proportion, percentages of postmenopausal women who experienced such change, either positive or negative, were calculated. To understand the extent of variation in participant responses, a range was used, whereas means, medians, and summarizing data statistics were used to report on the average experience and identify similarities and/or differences in average experience, if any, at different time points when testing occurred.

A participant information sheet, including a brief summary of the research study and its aims, clearly outlining the entire process in a language accessible to a lay audience was provided to all participants at baseline alongside a written informed consent form (according to the protocols of the National Ethics Committee for Clinical Studies), which they were asked to sign and return to confirm voluntary participation.

## Results

The postmenopausal women enrolled in this study had a mean age of 54 years (52-57 years old), described themselves as "female," were married, non-smokers, and declared consuming alcohol only socially or not at all. The mean BMI was 24.7 kg/m^2^. The baseline characteristics of study participants are shown in Table 1.

**Table 1:** Baseline characteristics of study participants mn: mean, y: years, no: number, kg: kilogram, m: meters, BMI: body mass index

Postmenopausal Women (n=108)	
Age (y, mean)	52-57 (54.0)
Gender - Female (no, %)	108 (100%)
Marital Status - Married (no, %)	108 (100%)
Smoking Status - Never Smoked (no, %)	108 (100%)
Alcohol Intake - None (no, %)	40 (37%)
Alcohol Intake - Low (no, %)	68 (63%)
BMI (mn, kg/m^2^)	24,7

Overall, findings indicate a considerable decrease in depressive symptoms in healthy postmenopausal women following the use of phytoestrogens over a six-month period.

When tested at baseline, the majority of postmenopausal women (87 out of 108) declared an experience of moderate depression, a smaller group (17 out of 108) had minimal or mild depression, while very few (4 out of 108) experienced severe symptoms.

There was a significant change, however, when testing occurred in Month 3, during which a sharp drop in the number of postmenopausal women with moderate depression was recorded (from 87 to 49 out of 108). In contrast, the number of women with minimal or mild depression appeared to be more than three times higher than what was reported at baseline (from 17 to 56 out of 108).

A similar pattern of change was observed during testing in Month 6. Upon study completion, the number of postmenopausal women experiencing moderate depression had further decreased (21 out of 108) whereas a significant increase in the number of women with minimal or mild depression was recorded (84 out of 108).

**Figure 2:**
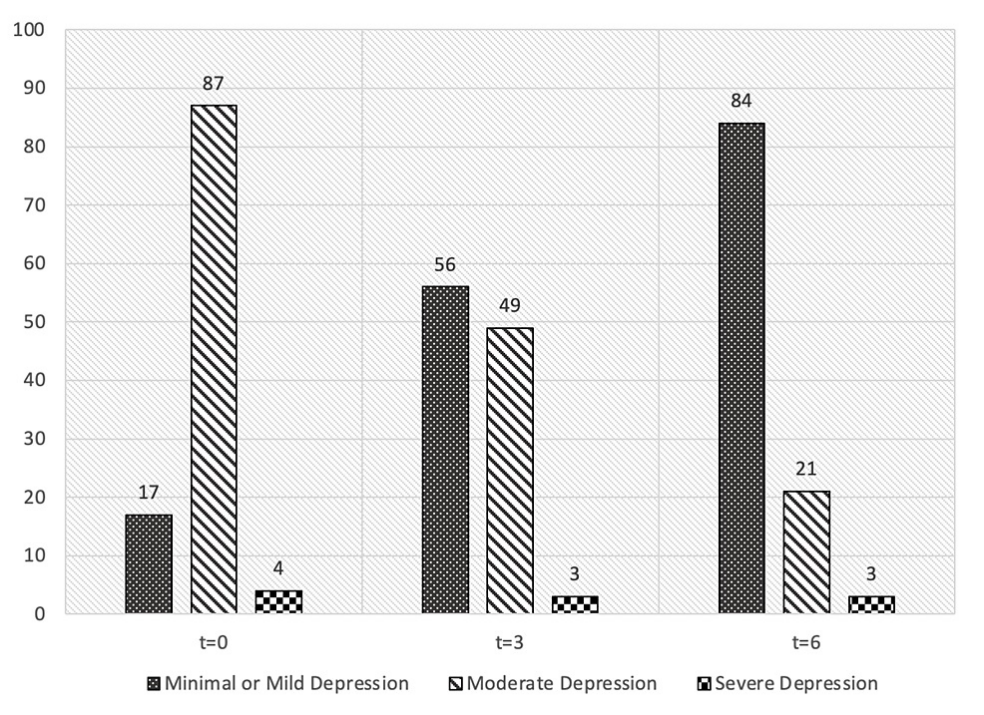
Reported outcome measures (N) across testing, Beck Depression Inventory-II

The changes in the number of participants allocated in each of the BDI II categories are depicted in Figure 2.

**Figure 3:**
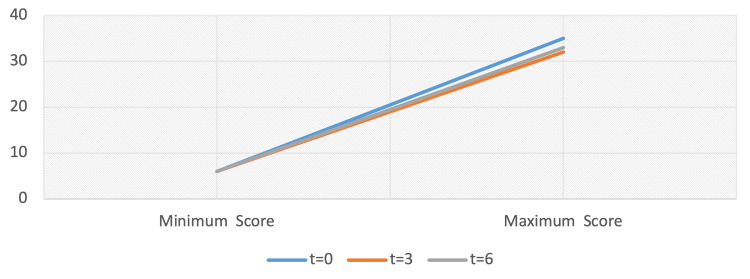
Range of individual outcome measures, Beck Depression Inventory-II

Minimal variation was observed when it came to minimum and maximum individual outcome measures, with cases in which scores were down to six out of 63 (at the lowest end of the BDI-II scale) and between 32 and 35 out of 63 (at the highest end of the BDI-II scale) being recorded at all testing times (range: 29 at t = 0; 26 at t = 3 and 27 at t =3) (Figure 3).

**Figure 4:**
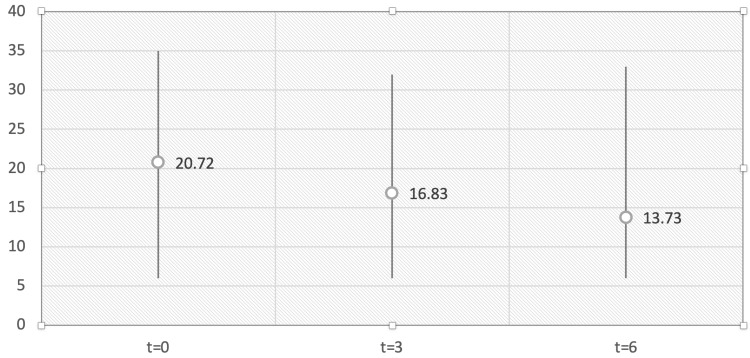
Mean of reported outcome measures, Beck Depression Inventory-II

The mean of reported outcome measures, however, seemed to be constantly and gradually decreasing from 20.72 at baseline to 13.73 upon study completion (Figure 4).

## Discussion

Overall, our findings indicate a considerable decrease in depressive symptoms in postmenopausal women following the use of phytoestrogens over a six-month period, suggesting that phytoestrogens could be used as an effective and reliable choice in order to manage and control post-menopausal depression.

Literature research on the efficacy of phytoestrogens in attenuating the psychological symptoms of menopause seemed to be quite inconclusive.

A systematic review published in 2017 demonstrated that phytoestrogens could improve anxiety and depression in menopausal women, the beneficial effects of which, however, could not be determined due to methodology issues, limited randomized controlled trials (RCTs), and small sample size [12]. Similarly, a meta-analysis published in 2018 revealed an association between phytoestrogen use and depression, demonstrating a significant reduction in depressive symptoms for menopausal women using phytoestrogens [13]. This meta-analysis though included only three articles, allowing for no definitive conclusions to be drawn regarding the effects of phytoestrogens on menopausal depression and, as such, for their use to remain controversial.

Findings from more recent systematic reviews indicate that some herbal medicines can positively contribute to relieving anxiety and depression and that phytoestrogens can have positive effects on the management of depressive symptoms in menopausal women, with more effects identified for larger dosages used, suggesting that phytoestrogens could be used to replace estrogens for the management and treatment of menopausal depression [14,15].

Some studies suggest that phytoestrogens may offer some relief from hot flashes and night sweats, but there is limited evidence of their ability to improve mood and decrease symptoms of depression during menopause [16].

Further studies are warranted to investigate the potential benefits of phytoestrogens in treating menopausal depression. Due to the high prevalence of depression during menopause and the potential risks associated with traditional hormone therapy, exploring alternative treatment options, such as phytoestrogens, is crucial for promoting mental health and quality of life in menopausal women [17].

The current study suffers from certain methodological limitations that need to be considered and addressed for future research. First, the small sample size of postmenopausal women enrolled in this study and the lack of a control group can affect the reliability of the results. Additionally, this is a single-center study, which further restricts findings in terms of generalization and comparison to a wider population. Also, the self-reported nature of outcomes using questionnaires could introduce bias and subjectivity. Moreover, depression evaluation was restricted to BDI-II with no other validated instruments or tools being used to assess and verify by comparing the results or account for other factors (e.g. quality of life) that might interfere with the outcome measures reported. Lastly, the correlation between sociodemographic characteristics, such as educational level and socioeconomic and relationship status, and depression were not explored in this study.

Future studies should, therefore, focus on exploring the effects of phytoestrogens on a wider population over a longer period of time and utilize objective measures to assess the efficacy of phytoestrogens in treating menopausal depression. To this end, planning and conducting multi-center longitudinal population-based studies would be beneficial. In addition, comparison study designs, allowing for both cases and controls to be included and studied, could offer better insights into the efficacy of phytoestrogen use on managing and reducing depressive symptoms in healthy postmenopausal women, reinforcing the reliability and accuracy of reported outcome measures. Moreover, future research should also consider different types of phytoestrogens, as well as the dosage and timing of administration, to determine their optimal effectiveness. Lastly, the correlation between sociodemographic characteristics, such as educational level and socioeconomic and relationship status, and depression were not explored in this study. Investigating the relationship between sociodemographic factors and depression during menopause could provide valuable insights into developing tailored treatment approaches for different populations.

## Conclusions

This study attempted to investigate the feasible effect of phytoestrogen administration on post-menopausal women experiencing depressive-like symptoms. The noteworthy decrease in symptom intensity in these cases implies that there might be a contribution of phytoestrogens to counteract the psychological symptoms of this period of a woman's life. Although the number of cases studied was relatively low in order to generalize the findings, the already known clinical suspicion has been deepened. In addition, our results encourage further studies to shed light on the course of factors that affect psychologically post-menopausal women and provide more information on a suitable treatment for patients.
